# Quantification of ATP and ADP levels in platelets from healthy human donors: a potential novel indicator of cardiovascular risk

**DOI:** 10.1186/1753-6561-9-S1-A34

**Published:** 2015-01-14

**Authors:** FM Buskandar, N Moran

**Affiliations:** 1 Molecular and Cellular Therapeutics, Royal College of Surgeons in Ireland, 123 St. Stephen’s Green, Dublin 2, Ireland

## Background

Understanding the biology of platelets is essential for the management of thrombotic diseases like stroke and cardiovascular disease. Platelets store ATP and ADP biomolecules and secrete them in response to thrombotic stimuli. However, patient-to-patient variability in storage of these biomolecules in platelets is poorly studied [1]. This study assesses ATP and ADP concentration in platelets and determines (1) the absolute values of these biomolecules per platelet, (2) the ratio of ATP and ADP in platelets and (3) the quantities released from dense granule storage sites in response to a discrete stimulus (Thrombin receptor activating peptide (TRAP); 10µM).

## Methods

A total of 21 healthy human donors were recruited with full ethical approval. Washed platelets (5x108/mL) were assessed with a commercial Enzlyte ADP/ATP Ratio Assay Kit to evaluate the total amounts of ATP and ADP. Briefly, platelets were stimulated with TRAP, or left untreated, in a 96-well plate containing a mixture of luciferase, substrate, co-substrate and/or ADP enzyme. Nucleotide levels were measured using a Wallac1420 multi-label counter. In parallel wells, detergent was added to platelet samples to allow determination of the total ATP and ADP load in the platelets.

## Results

The total levels of ATP and ADP biomolecules in platelets were 0.24 ±0.03 (nmoles per 108 platelets) and 0.23 ±0.04 (nmoles per 108 platelets), respectively. The ratio of ATP to ADP in platelets presented as 1.04: 1. In contrast, the amount of ATP and ADP secreted from the platelet’s dense granules in response to activation by TRAP was 2.1 ±0.51 (pmoles per 108 platelets) and 3.35 ±0.87 (pmoles per 108 platelets), respectively. The ratio of ATP to ADP, secreted from platelets upon activation, was 1: 1.59.

## Conclusions

Although platelets contain a baseline excess of metabolic ATP over ADP (Ratio= 1.04: 1), an excess of bioactive ADP is secreted in response to platelet activation. Moreover, we have also identified that there is a marked difference in the amount of ADP released by different donors. Therefore, understanding the intrinsic levels of ATP and ADP may lead to the identification of novel diagnostic method, which may assist in the early identification of patients at risk for thrombotic disorders. Future studies will compare ATP/ADP levels and ratios, between healthy patients and patients with thrombotic disease(s), in order to identify any differences between their total and released amounts of ATP/ADP and the ratio between them.
